# Cognitive constraints in bilingual processing—an entropy-based discrimination between translation and second language production

**DOI:** 10.3389/fpsyg.2025.1503147

**Published:** 2025-05-20

**Authors:** Chuanhong Tang, Danfeng Huang, Andrew K. F. Cheung

**Affiliations:** ^1^Guangzhou College of Commerce, Guangzhou, Guangdong, China; ^2^School of Foreign Languages, Guangdong Polytechnic Normal University, Guangdong, China; ^3^Hong Kong Polytechnic University, Kowloon, Hong Kong SAR, China

**Keywords:** bilingual processing, cognitive constraints, entropy, word n-gram, POS n-gram, text classification

## Abstract

This study investigates to what extent second language production (L2) and translational production are influenced by cognitive constraints due to their bilingual processing. Compared with monolingual production in first language (L1), the two bilingual productions are subject to the same cognitive constraints due to the co-activation of two linguistic systems and the language contact interference, though they involve two distinctive conceptualization stages, which may cause variations to their cognitive constraint. Entropy is utilized as an indicator of cognitive constraints in the study to illuminate how bilingual processing causes cognitive constraints to the two bilingual productions. Specifically, word and part-of-speech n-gram features are computed in the machine learning models to compare the three productions. The results show that L1 production could be effectively distinguished with the two bilingual productions, with L1 production exhibiting greater diversity and a more even distribution in most features than L2 production and translational production. This is clear evidence that both L2 production and translational production endure more cognitive load in the bilingual processing than L1 production. The results also reveal that L2 production and translational production could be discriminated against each other, with the former one exhibiting greater diversity and a more even distribution in most features than the latter one. These findings support the idea that both translators and L2 users belong to bilinguals affected by the cognitive load in bilingual processing although the two bilingual productions are constrained differently.

## 1 Introduction

This study empirically investigates the traces imposed by cognitive constraints in the two bilingual processing: L2 production and translational production. Although both translational production and L2 production operate under the constraints of bilingual processing, their production processes differ such that the former is interpretive and the latter is descriptive (Gutt, 2014; Heltai, 2010; Heltai and Lanstyák, 2018). The extent to which they are influenced by the cognitive constraint due to the bilingual processing remains unclear. This study addresses this issue by analyzing the entropy of part-of-speech (POS) n-gram and word n-gram, utilizing a data-mining approach to test a hypotheses.

L2 production and translational production share linguistic similarities because both are produced under the same cognitive constraints of bilingual processing, which involves the co-activation of two languages and language contact interference (De Groot and Christoffels, 2006; Lanstyák and Heltai, 2012; Toury, 2012). The simultaneous activation of two languages is thought to demand more cognitive resources than monolingual production (Jankowiak, 2021). These shared cognitive constraints may result in similar linguistic features arising in both L2 production and translational production. Simplification, for instance, is one of the most prominent shared traits (Lanstyák and Heltai, 2012; Liu et al., 2023), with researchers identifying at least two types: lexical simplification (Kwok et al., 2023) and syntactic simplification (Liu et al., 2023). Ivaska and Bernardini (2020) observed that L2 production and translational production exhibit several common characteristics, including a more formal style, fewer idiomatic expressions, fewer pronouns, and a greater use of explicit cohesive devices than L1 production. The two bilingual processing share other linguistic features, although these are often described using different terms due to the relative isolation between translation studies and second language acquisition (SLA) research (Kruger and Van Rooy, 2016). For example, in translation studies, the phenomenon of “explicitation” or “explicitness” is widely recognized (Englund Dimitrova, 2005; Gumul, 2006; Krüger, 2014; Olohan and Baker, 2000). A similar feature, termed “hyperclarity,” is identified in L2 production and refers to the tendency to increase formal explicitness (Andorno and De Cesare, 2017; Gullberg, 2009; Williams, 1987), providing further evidence of the linguistic parallels between L2 production and translational production.

The distinction between L2 production and translational production lies in the presence of an additional constraint in the latter: a pre-existing text (Lanstyák and Heltai, 2012). L2 production occurs in the absence of such a text, transferring mental representations directly into speech or writing. In contrast, typical translation or interpretation is inherently dependent on an antecedent text or spoken discourse. From a relevance-theoretic perspective, L2 production is a descriptive activity, whereas translational production is interpretive (Gutt, 2014; Heltai, 2010; Heltai and Lanstyák, 2018). This fundamental difference underscores that L2 production constitutes direct, non-mediated communication, whereas translational production involves mediation due to its reliance on a pre-existing text. Supporting this distinction, numerous studies have identified distinct characteristics in L2 production compared with translational production (Chen et al., 2024; Ivaska and Bernardini, 2020; Ivaska et al., 2022; Kajzer-Wietrzny and Ivaska, 2020; Kotze, 2022; Kruger and Van Rooy, 2016; Lanstyák and Heltai, 2012). For instance, Liu et al. (2023) used 14 syntactic complexity metrics to assess the complexity of L2 production and interpreted production, finding the two to be distinguishable. Their findings suggest that these forms of language production may be subject to differing cognitive constraints.

However, many linguistic features have only been utilized to differentiate L2, translational production and L1 productions from the perspective of language usage, overlooking the cognitive load behind the scene. For example, Kruger and Van Rooy (2016) implemented a multidimensional approach to identify parallels between languages produced by translational and L2 productions. These dimensions included informational versus involved production, explicit versus situation-dependent references, abstract versus non-abstract information, and online information elaboration; specific linguistic features, such as mean word length, emphatics, pronoun usage, modal verbs, and relative clauses, served as indicators of cognitive processing demands in bilingual contexts (Kruger and Van Rooy, 2016). Chen et al. (2024) differentiated L1, L2, and translational productions by examining syntactic complexity, using parameters such as clause length, sentence complexity, subordination, coordination, and specific syntactic structures. Ivaska and Bernardini (2020) distinguished L2 and translational productions in Finnish from form L1 production by analyzing POS distribution, verbal/clausal complexity, noun phrase complexity, and the use of proper nouns. The two bilingual productions and monolingual production can also be differentiated by lexical diversity, as word frequencies in production are influenced by the language proficiency of the L2 writer or the cognitive and sociolinguistic conditions affecting the translators. For instance, Kajzer-Wietrzny and Grabowski (2021) noted that translational production yielded a higher frequency of the most common word bi-gram patterns in both spoken and written language forms, compared with L1 production. The ongoing literature illustrates that there is a clear lack of research using features as indicator of cognitive load to delve into how L2 and translational productions are different from L1 production and also how the two bilingual processing are differently constrained. This methodological gap underscores the need for the current study.

In light of the above discussion, the extent to which L2 and translational productions resemble or differ from each other regarding their cognitive constraint remains inconclusive, underscoring the need for a feature measuring cognitive load by the traces left in language patterns. To address this research gap, this study uses entropy as a feature that measures the cognitive load by the diversity and distribution of POS n-gram and word n-gram. The remainder of this paper proceeds as follows. Section 2 reviews the literature. Section 3 outlines the methodology. Section 4 presents the results, which form the basis for the discussion in Section 5. Finally, Section 6 concludes the paper.

## 2 Literature review

This section reviews the literature on bilingual processing and the concept of entropy, providing the theoretical foundation for this study.

### 2.1 Bilingual processing

The idea of juxtaposing L2 and translational productions originates from studies on translation universals. For example, Blum and Levenston (1978) explored universal principles of lexical simplification across various linguistic contexts, such as translation and language learning, highlighting how strategies such as over-generalization, transfer, and circumlocution help convey meaning effectively despite vocabulary limitations. Subsequently, House and Blum-Kulka (1986) argued that explicitation, a translation universal, is likely to represent a common strategy in language mediation and should be examined across different types of interlanguages, including translated and learner language. Chesterman (1998, 2004) also emphasized the parallels between communication strategies, learning strategies, and translation strategies. Ulrych and Murphy (2008) reported that language used by non-native speakers and edited language share common features with translated texts. These studies actually call for a unified framework for the studies on L2 and translational productions.

Later, researchers began to examine L2 and translational production under the same framework of constrained communication, a concept referring to situations where language users face greater limitations on expression than in monolingual production (Lanstyák and Heltai, 2012). This framework is applicable to bilingual processing, as both L2 learners and translators navigate constrained language use. Lanstyák (2003) held the view that both L2 and translational productions involve the use of two languages, and translators are a subclass of bilinguals. Other research on bilingualism and cognitive processing has provided more evidence for Lanstyák (2003).

As bilingual processing, L2 and translational productions share common constraints, the most prominent of which is language contact interference (Lanstyák and Heltai, 2012). In both bilingual and translational productions, the interaction between two languages introduces a significant constraint: contact interference (De Groot and Christoffels, 2006; Lanstyák and Heltai, 2012; Toury, 2012). This interference often manifests as the transfer of linguistic elements from one language to another and the atypical use of structures in L2 (Weinreich, 1968). However, in bilingual processing, contact effects are not always limited to the abnormal use of linguistic items influenced by the other language in the speaker’s mind. They may also be “manifested in changes in the distribution of certain grammatical forms or lexical items under the influence of L2, or even their total avoidance” (Lanstyák and Heltai, 2012, p. 104). While these changes in distribution and diversity may be imperceptible to receivers, they can significantly alter the style of translation or speech, contributing to differences between the two bilingual productions and L1 production.

Another shared constraint is the cognitive load imposed by the co-activation of two languages in both translational and L2 productions (Berman and Ravid, 2008; Hopp, 2017; Jankowiak, 2021). During bilingual processing, suppressing the co-activation of two languages is challenging, because the representations and procedures that are used in L1 are highly accessible and rely on automatic routines (Bergmann et al., 2015). In L2 production, bilingual speakers may unconsciously form concepts or structures in their native language, leading to the automatic activation of their first language, which then influences the production of speech or written language. Whether a bilingual speaker attempts to suppress or manage this co-activation, it requires more cognitive resources than monolingual tasks. In translational production, this co-activation is more overt, as both L1 and L2 are actively used. The presence of these two constraints in both L2 and translational production inevitably leaves traces in language use.

Translational production may be more constrained than L2 production due to the additional constraint imposed by its unique mode of language use. The primary distinction between the two types of bilingual processing stems from the nature of the task: L2 production involves independent text production, whereas translation involves dependent text production (Lanstyák and Heltai, 2012). In relevance-theoretic terms, bilingual language use is descriptive, whereas translation is interpretive (Lanstyák and Heltai, 2012). This characterization of translation as interpretive language use is closely tied to its definition as an act of explanation or interpretation (Bühler, 2002; Chesterman, 2008). From this perspective, translational production involves three key steps: comprehension, deverbalization, and re-expression in the target language (Lederer and Larché, 2014). These steps involve interpreting or explaining the ideas or intentions behind a source text, rather than merely translating its lexical semantics. Consequently, the pre-existing text or speech acts as a constraint on translational production, limiting translators’ freedom to choose words and sentence structures and often confining them to the information and sometimes even the structure of the original text. In contrast, L2 production does not require a pre-existing text or speech. Speakers or writers in L2 production are relatively unconstrained in their choice of words and grammatical structures, allowing them to freely express their ideas. This freedom in L2 production, compared with the constraints inherent in translation, makes L2 production less constrained than translational production. This difference in the degree of constraint is a key factor distinguishing L2 production from translational production.

### 2.2 Entropy: a feature for measuring cognitive load

To address the gap of comparing bilingual productions and monolingual production from the perspective of cognitive load, this study proposes the use of entropy as a feature to highlight the informativeness of a language text. Informativeness, recognized as a critical global feature of text (Berman and Ravid, 2008; Khairova et al., 2019; Shams, 2014; Wu and Giles, 2013), has been utilized as a holistic linguistic feature in SLA (Ehret and Szmrecsanyi, 2019; Kutay, 2024; Osborne, 2011) and translation studies (Espunya, 2007; Lin and Liang, 2023; Wong and Kit, 2011). Entropy is a key concept in information theory, originally developed for telecommunications and cryptography applications (Lin and Liang, 2023). It quantifies the maximum amount of information that can be transmitted through specific channels (Shannon, 1948, 1949) and indicates the “average information content and the average uncertainty of a discrete variable” (Lin and Liang, 2023, p. 3). As a holistic metric, entropy mitigates bias by considering all elements within a given entity.

In the context of language texts, entropy can be used as a feature implying the cognitive load behind the scene (Wei, 2021, 2022). Carl et al. (2019) in his “systems theory perspective,” mentioned that the process of human translation can be viewed as a complex arrangement of interconnected systems for translating words and phrases, which work together and form organized structures that disperse energy and entropy is defined as the internal order of these word translation systems. This is more likely to describe entropy as a mental process, as echoed by Wei (2021). Wei (2021) explained the nature of entropy as probability distribution and decision uncertainty and how entropy mirrors cognitive resource allocation among competing translation options. Entropy could be used to “describe and explain cognitive activities when mental states transition between one another during lexical activation and selection” (Wei, 2022, p. 77). In bilingual processing, be it L2 or translational production, there is always word selection “where there is continual shift of cognitive resource allocation as mental states transition from one towards another” (Wei, 2022, p. 77). Following the conceptual exploration, the amount of cognitive load needed in the process can thus be quantified via the reduction of entropy (Wei, 2022). Specifically, if a bilingual experiences more cognitive load imposed by co-activation of two languages or language contact interference, he will have less efforts allocated into word and POS selection during language production since the cognitive resources are limited, where the entropy will be reduced since the probability of changes will decrease. Therefore, entropy is inversely proportional to the cognitive load that a bilingual is receiving in bilingual processing.

In the context of language texts, this study proposes to utilize the entropy of word n-gram and POS n-gram to reveal the non-uniform distribution within a text. Word n-gram are simply consecutive sequences of “n” words from a text. For instance, in the sentence, “Languages and cultures are inseparable,” the word tri-gram would include sequences such as (“Language,” “and,” “culture”) and (“and,” “culture,” “are”) and the word bi-gram would include sequences such as (“Languages,” “and”) and (“and,” “culture”). In contrast, POS n-gram take it a step further by organizing words into their grammatical categories (e.g., nouns, verbs, and adjectives) and then forming sequences of these parts of speech. Using the same example sentence, after POS tagging, the sequence would be represented as (“noun,” “conjunction,” “noun,” “verb,” “adjective”) and the POS bi-gram would be represented as (“noun,” “conjunction”) and (“conjunction,” “noun”), and so on. The probability of a word or word n-gram and a certain POS or POS n-gram is inversely proportional to the overall entropy of the text (Chen et al., 2017). Consequently, higher entropy values indicate greater information content, whereas lower values suggest less (Chen et al., 2017). The entropy-based method has been extensively applied and has demonstrated its effectiveness in quantitative research, including text classification (Chen et al., 2017; Largeron et al., 2011; Wang et al., 2024), translation studies (He et al., 2010; Liu et al., 2022), and SLA (Flanagan and Hirokawa, 2015).

This study investigates to what extent second language production (L2 production) and translational production are influenced by cognitive constraints due to their bilingual processing. It uses the written texts of L1, L2 and translational productions as the research objects to explore the traces in language usage imposed by cognitive constraints in bilingual processing. Six features (entropy of word uni-gram, word bi-gram, word tri-gram, POS uni-gram, POS bi-gram, and POS tri-gram) are used as the metric to investigate the cognitive load. Specifically, this study seeks to answer the following research questions:

**RQ1:** Can the quantitative features of entropy for word n-gram and POS n-gram effectively distinguish between L1, L2 and translational productions?

**RQ2:** If yes for RQ1, which two features contribute the most to this classification, and which two features contribute the least?

**RQ3:** In what ways do L2 and translational productions resemble and differ from each other?

**RQ4:** What underlying factors account for the differences and similarities between L2 and translational productions?

## 3 Methodology

This section elaborates on the corpus used in the study, the methods used to calculate entropy, and the machine learning models used for text differentiation.

### 3.1 Corpus composition

To compare the entropy of word n-gram and POS n-gram across the three languages of L1, L2 and translational productions, a parallel corpus consisting of real-world political news was constructed. The corpus comprised three types of texts:

1. **Translated language:** A total of 420 editorial articles, published between June 1, 2022 and May 31, 2024, were randomly collected from the Global Times, a prominent news website that publishes content in both Chinese and English. All of the English-language articles on this platform are translations from original Chinese texts.
2. **L2:** This sub-corpus included 420 randomly selected editorial articles, also published between June 1, 2022 and May 31, 2024, sourced from the editorial module of China Daily. These articles are authored by Chinese reporters who use English as a foreign language.
3. **L1:** Comprising another 420 randomly selected editorial articles published between June 1, 2022 and May 31, 2024, this sub-corpus was derived from The Guardian, a platform known for its English articles written by native speakers.

All three platforms publish editorial articles commenting on international events, enhancing the comparability of the three sub-corpora.

To mitigate potential bias related to text size, the Python scripts used in this study processed only the first 500 words of each article for entropy calculations. An overview of the corpus is provided in Table 1.

**Table 1:** Overview of the corpus.

Sub-corpora	Text count	Sources	To-be-processed text size
Translated language	420	*Global Times* (from June 1, 2022 to May 31, 2024)	210,000 (the first 500 words of every text)
L2	420	*China Daily* (from June 1, 2022 to May 31, 2024)	210,000 (the first 500 words of every text)
L1	420	*The Guardian* (from June 1, 2022 to May 31, 2024)	210,000 (the first 500 words of every text)

### 3.2 Entropy calculation methods

The concept of entropy in a text is a feature of the uniformity of the distribution of certain language units within it, based on the idea of information entropy (Shannon, 1948). Entropy is calculated as follows:


(1) $$H=-\sum_{i=1}^{n}p(i)\log_{2}p(i)$$

where *H* represents entropy, n represents the total number of word n-gram or POS n-gram types present in the text, and *p*(*i*) represents the relative frequency of the *i*-th type of word n-gram or POS n-gram in the text.

As shown in the above formula, the first step is to calculate *p*(*i*):


(2) $$p(i)=\frac{f(i)}{N}$$

where *f*(*i*) represents the frequency of the *i*-th type of word n-gram or POS n-gram and *N* represents the total number of word n-gram or POS n-gram that appear in the text. A more detailed explanation of entropy is provided for the following example sentence from the L2 sub-corpus:

*The two sides will hold the first meeting of the China–U.S. intergovernmental dialogue on artificial intelligence, and continue various other exchange mechanisms.*

The corresponding POS of the above example sentence is as follows: Det, Num, Noun, Aux, Verb, Det, Adj, Noun, Prep, Det, Noun, Adj, Noun, Prep, Adj, Noun, Conj, Verb, Adj, Adj, Noun, Noun. As the total number of word tokens is 22, *N* equals 22. The word “the” appears three times and the word “sides” appears one time. p(the) and p(sides) can be calculated as follows:


$$p(\text{the})=\frac{3}{22}\;p(\text{sides})=\frac{1}{22},\mathrm{etc}$$

The next step is to calculate the p(i) values of all word types. The entropy of word uni-gram for the whole sentence can be calculated as follows: $H(T)=-(\begin{array}{l} \frac{3}{22}\log_{2}\frac{3}{22}+\frac{1}{22}\log_{2}\frac{1}{22}+\\ \frac{7}{22}\log_{2}\frac{7}{22}\ldots +\frac{1}{22}\log_{2}\frac{1}{22} \end{array})\approx 4.46\;\text{bits}/\text{word}$ .

This formula can be applied to POS uni-gram to calculate the POS entropy of this example sentence: $H(T)=-(\begin{array}{l} \frac{3}{22}\log_{2}\frac{3}{22}+\frac{1}{22}\log_{2}\frac{1}{22}+\\ \frac{1}{22}\log_{2}\frac{1}{22}\ldots +\frac{1}{22}\log_{2}\frac{1}{22} \end{array})\approx 2.97\;\text{bits}/\text{word}$ .

As indicated by the formulas, a higher entropy of word n-gram in a given text suggests a more uniform distribution of different word types, meaning that word types are less likely to repeat, which results in greater diversity and informativeness. Conversely, if a text predominantly features a limited number of word types appearing frequently, its word n-gram entropy will be lower. This principle also applies to POS n-gram.

The three sub-corpora were initially parsed using Python scripts through the Natural Language Toolkit (NLTK), a powerful library for processing human language data. The NLTK provides a comprehensive suite of tools for text processing, categorization, tagging, semantic reasoning, tokenization, parsing, and various other language-processing tasks (Hardeniya et al., 2016). Subsequently, the first 500 words and 500 POS tags from each text were used to calculate the entropy of both word n-gram and POS n-gram. All six features for each text were stored in an .xls file for subsequent analysis.

### 3.3 Text classification models and other analysis tools

The major classification algorithms for text classification include logistic regression, decision trees, random forests, support vector machines (SVMs), k-nearest neighbors (k-NN), and naïve Bayes (Kutay, 2024). However, it is crucial to carefully select the most appropriate classification methods for specific research purposes (Jing and Yao, 2023). Five machine learning classifiers were utilized in this study: random forests, SVMs, logistic regression, k-NN, and decision trees. The area under the curve (AUC) values and accuracy of each classification model were compared to identify the model yielding the best classification performance for this study.

SVMs are a class of generalized linear classifiers defined as systems that use a hypothesis space of a linear function in a high-dimensional feature space. These classifiers are trained with algorithms grounded in optimization theory that implement learning biases derived from statistical learning theory (Jakkula, 2006). Since their introduction, SVMs have been used in applications across various fields, including cancer genomic studies (Huang et al., 2018) and image classification (Chandra and Bedi, 2021). SVMs have also demonstrated their effectiveness in text classification (Colas and Brazdil, 2006; Goudjil et al., 2018; Liu et al., 2010; Selva Birunda and Kanniga Devi, 2021), leading to their use in comparing constrained and non-constrained languages (Hu and Kübler, 2021; Liu et al., 2022). As such, SVMs were selected for implementation in this study.

Random forests, introduced by Breiman (2001), represent a family of classification methods that operate by constructing a multitude of decision trees during the training phase. The final output is determined by either the mode of the classes (classification) or the mean prediction (regression) of the individual trees. This method has also been applied in various research contexts, including compound classification (Svetnik et al., 2003), remote sensing (Belgiu and Drăguţ, 2016), and survival analysis (Rigatti, 2017). Notably, random forests have been used in linguistic studies (Levshina, 2021; Maitra et al., 2015; Th Gries, 2020), making them suitable for this study.

Logistic regression is a statistical model used for binary classification tasks, predicting the probabilities of different possible outcomes for a categorical dependent variable based on several independent variables (LaValley, 2008; Nick and Campbell, 2007). This method has been widely applied in various fields, including healthcare (Issitt et al., 2022; Panda, 2022), facial recognition (Khalajzadeh et al., 2014; Singh and Singh, 2017), and text classification (Bahtiar et al., 2023; Shah et al., 2020). This study adopted logistic regression for its robust binary classification capabilities.

k-NN is a commonly used technique for text classification in quantitative linguistics (Tay, 2024). In k-NN text classification, features are viewed as dimensions that locate the corresponding data point (text) in Euclidean space. The classification of an unlabeled text is determined by consulting its k-nearest neighbors; the text type that appears most frequently among these neighbors is assigned to the unlabeled text. k-NN plays a significant role in various areas, including SLA (Altay, 2022) and text classification (Ababneh, 2019; Bhavani and Kumar, 2021). Thus, k-NN was used in this study.

Decision trees are algorithms that predict outcomes based on simple decision rules inferred from data features. They work by recursively splitting the data into branches based on the most informative feature, creating a tree structure. Decision tree algorithms have been widely applied in various fields, including medical diagnosis (Azar and El-Metwally, 2013), text classification (Charbuty and Abdulazeez, 2021), and linguistic studies (Kotani and Yoshimi, 2017). Therefore, decision trees were also used in this study.

To visually analyze the similarities and differences between L1, L2, and translational production, t-distributed stochastic neighbor embedding (t-SNE) visualization was used. t-SNE is a nonlinear dimensionality reduction technique that effectively reduces multiple dimensions into two principal dimensions, preserving the numerical features of the original data and exceling at visualizing high-dimensional data by optimizing the relative positions of data points in a lower-dimensional space to reflect their local structures (Van Der Maaten, 2014; Van der Maaten and Hinton, 2008). Additionally, a detailed analysis of how translated language and L2 compare and contrast was conducted using IBM SPSS 26, with a focus on six features and L1 serving as a baseline.

The .xls file containing the six features for each text was utilized to train and test the corresponding classifiers across the aforementioned five classification models. For each model, the data in the .xls file were randomly split into two subsets: 75% for training the classifier and the remaining 25% for testing the trained classifier. The study was designed to compare L2 versus L1, L2 versus translated language, and L1 versus translated language. Consequently, for each model, three classifiers were trained to classify L2 versus L1, L2 versus translated language, and L1 versus translated language, resulting in three AUC scores and three accuracy scores for each model. The mean AUC and accuracy of the three classifiers were used as the final AUC and accuracy metrics for the specific model, allowing for the selection of the model with the best performance (Liu et al., 2022).

## 4 Results

As shown in Table 2, all five classification models produced excellent results, with the SVM model achieving the highest performance and the decision tree model ranking lowest. While there is no universal acceptance threshold for a classification model’s accuracy, this study adopted an accuracy score of 0.5 as the minimum acceptable threshold, as suggested by Lapshinova-Koltunski (2022). All of the models exceeded this threshold, with accuracy scores ranging from 0.78 to 0.82. Addressing RQ1, these results indicate that the five models are capable of effectively distinguishing between L2, L1, and translated language based on the following six features of entropy for word n-gram and POS n-gram: word uni-gram, word bi-gram, word tri-gram, POS uni-gram, POS bi-gram, and POS tri-gram.

**Table 2:** The average AUC and average accuracy for five models.

Classification model	Average AUC	Average accuracy
SVMs	0.8789	0.82
Logistic regression	0.8773	0.82
K-NN	0.8770	0.81
Random forest	0.8551	0.79
Decision trees	0.7772	0.78

The following results and analysis addressed RQ2. Given that the SVM model had the highest average AUC and accuracy, a more detailed analysis was conducted using its output. Table 3 presents the coefficients of the six features for the SVM classifier trained to distinguish between L2 and translated language. No predefined range for the coefficients was set; instead, the absolute value of a coefficient indicates the importance of the corresponding feature in differentiating the two types of texts. A higher absolute value suggests greater significance in classification. Additionally, features with positive coefficients are more likely to predict Label 1 (i.e., L2 in this case), whereas those with negative coefficients are more likely to predict Label 2 (i.e., translated language in this case). The results in Table 3 show that POS bi-gram and word tri-gram were the most crucial features for distinguishing L2 from translated language. Conversely, POS uni-gram and word uni-gram had minimal impact on the classification. This suggests that L2 and translated language differ significantly in POS bi-gram and word tri-gram, but they share similarities in POS uni-gram and word uni-gram. It is important to note that the coefficient value reflects the importance of a feature for classification, not the scale of the feature’s value.

**Table 3:** Feature coefficients for L2 vs. translated language.

Feature	Coefficient	Important rank
POS bi-grams entropy	1.31993	1
Word tri-grams entropy	−0.91945	2
POS tri-grams entropy	−0.53208	3
Word bi-grams entropy	−0.45514	4
POS uni-gram entropy	−0.33885	5
Word uni-gram entropy	0.26481	6

As shown in Table 4, the classifier trained to classify L2 versus L1 produced the six feature coefficients, with word uni-gram entropy and POS uni-gram entropy occupying the first and second positions in the ranking. This implies that when the task is to distinguish L2 and L1 texts, it is wise to consult the word uni-gram entropy and POS uni-gram entropy. In contrast, L2 and L1 diverged the least in two features: POS tri-gram entropy and word bi-gram entropy.

**Table 4:** Feature coefficients for L2 vs. L1.

Feature	Coefficient	Important rank
Word uni-gram entropy	2.64516	1
POS uni-gram entropy	−2.02933	2
Word tri-grams entropy	1.30479	3
POS bi-grams entropy	0.91473	4
POS tri-grams entropy	0.49509	5
Word bi-grams entropy	−0.41073	6

Similarly, Table 5 demonstrates the feature coefficients for translated language versus L1. The two features that had the greatest effect on differentiating translated language from L1 were word uni-gram entropy and POS uni-gram entropy. In contrast, the two features that had the weakest effect on differentiating translated language from L1 were word bi-gram entropy and POS bi-gram entropy.

**Table 5:** Feature coefficients for translated language vs. L1.

Feature	Coefficient	Important rank
Word uni-gram entropy	−2.27742	1
POS uni-gram entropy	1.37433	2
Word tri-gram entropy	−1.17192	3
POS tri-gram entropy	−0.70293	4
Word bi-gram entropy	0.14000	5
POS bi-gram entropy	−0.07652	6

Further comparing the results in Tables 4, 5 would help identify the similarities between L2 and translated language, with L1 as the baseline. It is clear from both tables that the features that contributed most to the classification between L2 versus L1 and between translated language versus L1 were the same: word uni-gram entropy, POS uni-gram entropy, and word tri-gram entropy. These two classifications were run with L1 as the baseline, demonstrating that L2 and translated language were somehow similar to each other when compared with L1. Overall, these findings addressed RQ2.

**Figure 1:**
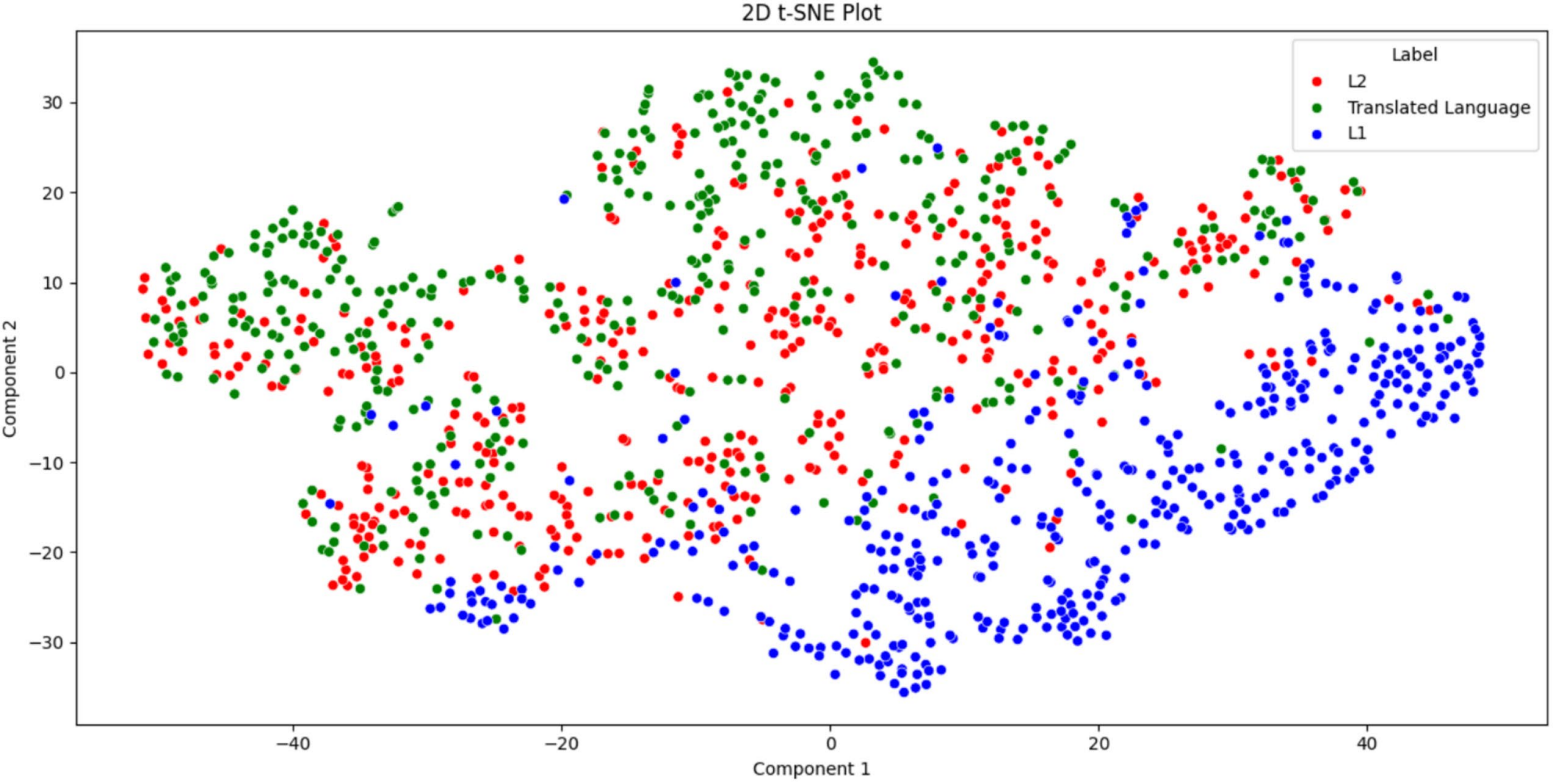
t-SNE scatter plot for L1, L2, and translated language.

The following results and analysis addressed RQ3. Figure 1 presents the t-SNE scatter plot for L1, L2, and translated language. L1 texts predominantly occupy the lower-right section, L2 texts are concentrated in the middle, and translated texts cluster in the upper-left section. However, L2 texts and translated texts do share some overlapping areas. In a t-SNE scatter plot, the proximity of two dots (representing texts) indicates their similarity, whereas greater distance implies greater dissimilarity. Based on Figure 1, it can be concluded that L1, L2, and translated language are distinguishable from each other. However, L2 texts and translated texts exhibit fewer differences compared with L1. Additionally, the plot suggests that L2 plays a “mediating role” between translated language and L1, as most L2 texts are positioned between the clusters of L1 and translated texts. This indicates that L2 shares characteristics with both L1 and translated language, although it remains a distinct variety.

The above conclusion could also be echoed by the “average distance to the decision boundary” produced by the SVM model. In SVMs, the average distance to the decision boundary measures the mean distance between all of the dots (texts) and the separating hyperplane in Euclidean space, with a greater mean distance suggesting a more efficient classifier for the two texts to be classified. Therefore, the average distance to the decision boundary could also be used as the extent to which the two types of texts differ.

The results in Table 6 show that translated language versus L1 was the most heterogeneous text pair, with the average distance to the decision boundary reaching 3.0397. In contrast, L2 versus translated language was the least heterogeneous text pair, with L1 as the baseline. In other words, L2 and translated language were less different from each other compared with L1.

**Table 6:** The average distance to decision boundary.

Text pair	Average distance to decision boundary
L2 vs. L1	2.9942
Translated language vs. L1	3.0397
L2 vs. Translated language	0.8607

**Figure 2:**
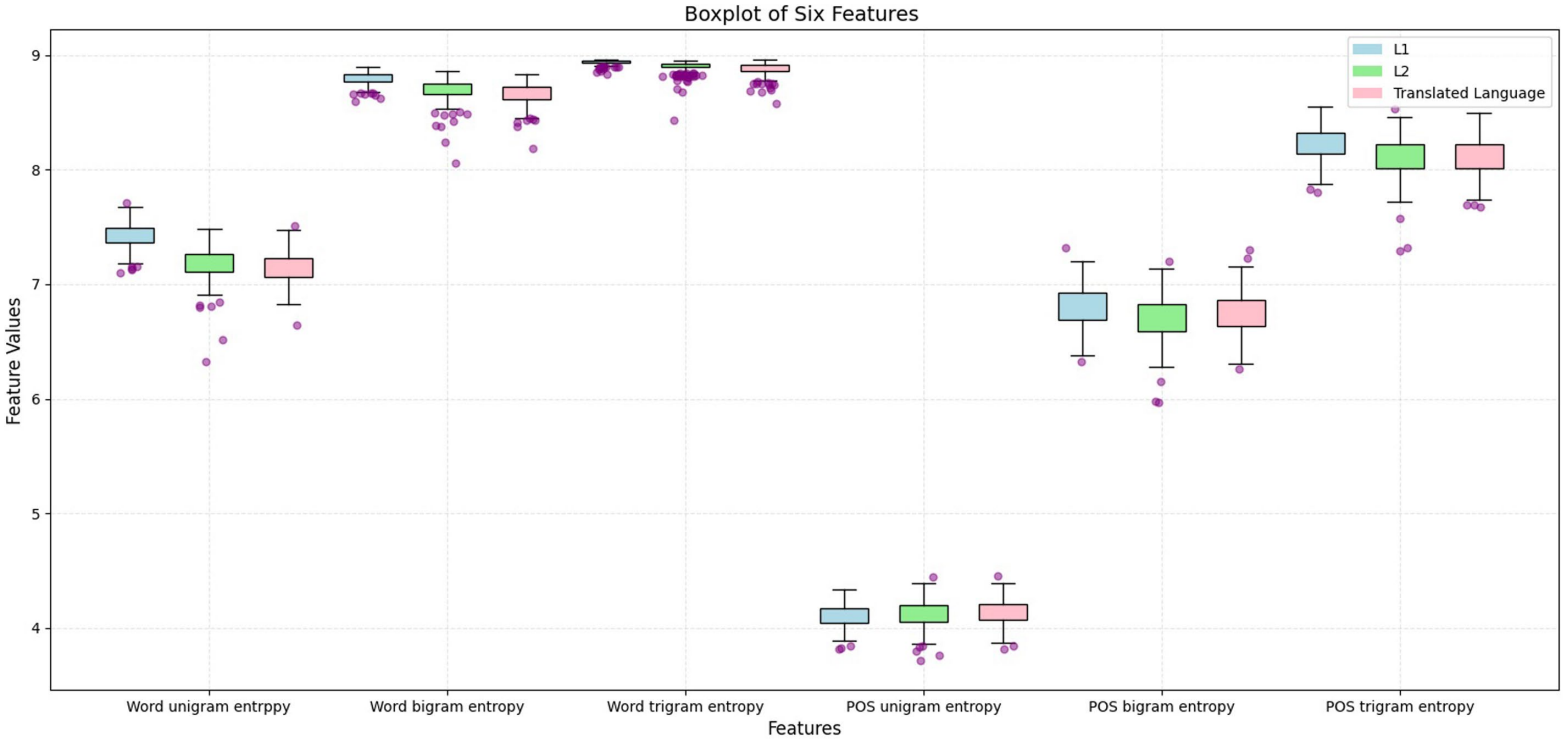
Boxplot for the six features of L1, L2, and translated language.

However, the features in which L2 versus translated language were less different, with L1 as the baseline, remained unknown and were further explored using the following process. IBM SPSS 26 was used to conduct an analysis of variance (ANOVA), and Figure 2 and Table 7 were created to determine how L2, L1, and translated language differed from each other in terms of the six measured features.

**Table 7:** The ANOVA test results.

Dependent variable	(I) type	(J) type	Mean difference (I–J)	Sig.
Word unigram entropy	L2	TL	0.032411566671071*	0.000
	L2	L1	−0.248763992043899*	0.000
	TL	L1	−0.281175558714970*	0.000
Word bigrams entropy	L2	TL	0.040867662846681*	0.000
	L2	L1	−0.098431948220695*	0.000
	TL	L1	−0.139299611067376*	0.000
Word trigrams entropy	L2	TL	0.024773375833389*	0.000
	L2	L1	−0.033783914104566*	0.000
	TL	L1	−0.058557289937955*	0.000
POS unigram entropy	L2	TL	−0.017911337841277*	0.009
	L2	L1	0.018491554925555*	0.007
	TL	L1	0.036402892766832*	0.000
POS bigrams entropy	L2	TL	−0.036005470469853*	0.002
	L2	L1	−0.091149260124419*	0.000
	TL	L1	−0.055143789654566*	0.000
POS trigrams entropy	L2	TL	−0.009907298	0.328
	L2	L1	−0.122119242017835*	0.000
	TL	L1	−0.112211944428223*	0.000

* is a mark in ANOVA test meaning there is significant difference between the two compared data.

Figure 2 ranks L1, L2, and translated language according to the mean values of six features, as follows:

- Word uni-gram entropy: L1 > L2 > Translated language
- Word bi-grams entropy: L1 > L2 > Translated language
- Word tri-grams entropy: L1 > L2 > Translated language
- POS uni-gram entropy: L1 < L2 < Translated language
- POS bi-grams entropy: L1 > Translated language > L2
- POS tri-grams entropy: L1 > Translated language > L2

These rankings show that L1 generally exhibited higher diversity and a more even distribution in five of the six features compared with L2 and translated language. The only exception was POS uni-gram entropy, where L2 and translated language outperformed L1, indicating a more varied and more evenly distributed use of POS uni-gram in the two constrained languages.

The consistent intermediate ranking of L2 for four of the six features further reinforced its mediating role between L1 and translated language, echoing the observations made in Figure 1, where L2 texts tend to occupy the central region of the scatter plot. Furthermore, L1 never ranked in the middle, underscoring its distinct position relative to L2 and translated language. Both L2 and translated language exhibited similar tendencies in terms of word n-gram and POS n-gram diversity and distribution. Specifically, in five features, both L2 and translated language ranked lower than L1, whereas in the case of POS uni-gram entropy, they ranked higher. This consistent alignment suggests a strong resemblance between L2 and translated language, irrespective of the direction of the entropy values. Overall, these results addressed RQ3.

## 5 Discussion

This study used entropy to assess the diversity and distribution of word n-gram and POS n-gram across three language types: two languages by bilingual processing (L2 and translational productions) and one monolingual language (L1 production). The six features were then analyzed using five classification models—SVMs, decision trees, random forests, k-NN, and logistic regression—to determine whether these language types could be distinguished based on the selected features. IBM SPSS 26 and t-SNE visualization were utilized to conduct a detailed analysis. The shared constraints inherent in bilingual processing were found to contribute to the differentiation between the two bilingual productions and the monolingual production. With the results addressing RQ1, RQ2, and RQ3 in Section 4, the following discussion addresses RQ4.

### 5.1 Similarities between the languages by two bilingual productions

The shared cognitive constraints inherent in L2 production and translation contribute significantly to the observed similarities between the languages by L2 and translational productions. Scholars have long speculated that translation universals may also be applicable to broader contexts of constrained communication. For instance, Kotze (2022) suggested that the characteristics typically associated with translation could be more broadly understood as features or “universals” of language mediation, language contact, bilingual or multilingual discourse production, and other forms of constrained communication. Kruger and Van Rooy (2016) pointed out that language production in translation is cognitively constrained due to the activation of bilingual language systems—an aspect that similarly affects L2 production. Furthermore, Heltai and Lanstyák (2018) argued that L2 and translational productions share similarities in their linguistic processes, as both are conducted by bilingual individuals whose mental representations and control of their two language systems are alike. Considering that translators can be viewed as a subset of bilingual speakers (Andorno and De Cesare, 2017; Heltai, 2010; Lanstyák, 2003), it is reasonable to assert that L2 and translational productions are influenced by common constraints. These constraints include the high cognitive load imposed by the simultaneous activation of two languages and the strict adherence to perceived standard language norms (Kruger and Van Rooy, 2016). As a result, these shared constraints affect language production in both L2 and translational production, leading to similar linguistic patterns, such as reduced lexical diversity (Kajzer-Wietrzny and Ivaska, 2020), lexical simplification (Blum and Levenston, 1978; Niu and Jiang, 2024), and lower POS diversity (Ivaska and Bernardini, 2020) in constrained languages.

As demonstrated in Section 4, the similarities between the two languages by L2 and translational productions—are evident in the results. In the t-SNE visualization, these two languages shared a significant portion of Figure 1, indicating a notable degree of similarity. Additionally, the L2 versus translated language text pair exhibited the smallest average distance to the decision boundary, compared with the other pairs (L2 vs. L1 and translated language vs. L1). This suggests that L2 and translated language are more alike, with L1 serving as the baseline. Further evidence of these similarities can be seen in their parallel trends in word n-gram and POS n-gram distribution and diversity, as depicted in Figure 2. Furthermore, the L2 versus translated language pair demonstrated the smallest mean differences among the three text pairs, reinforcing the notion of their similarity. Finally, the fact that L2 and translated language could not be distinguished based on POS tri-gram entropy suggests that they share similarities in the diversity and distribution of POS tri-gram.

### 5.2 Differences between the languages by two bilingual productions

The additional cognitive constraint in the translation process accounts for the differences between the languages by translational and L2 productions. While both translational and L2 productions involve the use of two languages, translation differs fundamentally from L2 production. The key distinction lies in the nature of language use: ordinary L2 production typically involves descriptive language, where individuals use language to express thoughts or ideas from their minds. In contrast, translation involves interpretive language use, where translators produce language in response to a pre-existing text or speech, treating language as a tool for interpretation (Heltai and Lanstyák, 2018). In typical translation contexts, translators are cognitively constrained by the source text or speech, as they are guided by translation ethics, norms, or directives from translation activity organizers (Chesterman, 1993). Such adherence to the source material can influence the language used by translators, often resulting in the phenomenon known as translation universals (Chesterman, 2004; Halverson, 2003; House, 2008; Lanstyák and Heltai, 2012; Mauranen and Kujamäki, 2004). One specific outcome of this influence is lexical simplification, where the translated text tends to use simpler vocabulary than the original (Blum and Levenston, 1978; Kajzer-Wietrzny and Ivaska, 2020).

The additional cognitive constraint of the translational production compared with L2 production (Lanstyák and Heltai, 2012) is evident through the “middle” role played by L2 between the languages by translational and L1 productions. As shown in Table 6, the average distance to the decision boundary was the largest for translated language versus L1 at 3.0397, with L2 versus L1 occupying an intermediate position and L2 versus translated language having the smallest distance. This positioning indicates that L2 serves as a “middle” role, with translated language and L1 at opposite ends of the spectrum. Further evidence of this “middle” role can be seen in Figure 2, where L2 consistently ranks in the middle position for four of the six features measured. Given that both L2 and translated language exhibit similar tendencies in terms of word n-gram and POS n-gram diversity and distribution, and considering L2’s intermediary role, the following deduction can be made: the shared cognitive constraints of translational and L2 productions lead to reduced diversity in word uni-gram entropy, word bi-gram entropy, word tri-gram entropy, POS bi-gram entropy, and POS tri-gram entropy, with an increased diversity in POS uni-gram compared with L1 production. However, due to the additional cognitive constraints inherent in the translation process, translated language demonstrates even lower diversity in word uni-gram entropy, word bi-gram entropy, word tri-gram entropy, POS bi-gram entropy, and POS tri-gram entropy, and greater diversity in POS uni-gram compared with L2.

## 6 Conclusion

Entropy features—word n-gram entropy and POS n-gram entropy—were used across five classification models to distinguish L2, L1, and translational productions. The findings indicate that the three languages by L2, L1, and translational productions can be differentiated based on these combined features. L1 production demonstrated greater diversity and a more even distribution across word uni-gram entropy, word bi-gram entropy, word tri-gram entropy, POS bi-gram entropy, and POS tri-gram entropy, but it exhibited less diversity and a less even distribution for POS uni-gram entropy. In contrast, L2 and translational productions shared similar tendencies in word n-gram and POS n-gram diversity and distribution due to their shared cognitive constraints as bilingual processing. Despite their similarities, L2 and translational productions could still be distinguished from each other in five of the six features, with POS tri-gram entropy being the exception. Notably, L2 production appears to play a “middle” role between L1 and translational productions, with translational production experiencing additional cognitive constraints.

These empirical findings support the hypothesis that translational production is a subset of bilingual processing, though distinguished from L2 production by its additional cognitive constraints. However, this study has some limitations. While evidence was provided for the existence of additional constraints in the translation process, the analysis was limited to word n-gram entropy and POS n-gram entropy, excluding other holistic features, such as syntactic dependency tree entropy. Additionally, the study focused solely on editorial news texts; future research should explore a wider range of genres to further validate this study’s findings.

## Data Availability

The datasets presented in this study can be found in online repositories. The names of the repository/repositories and accession number(s) can be found in the article/supplementary material.
